# Supplementary material for: Effect of Time-Dependent Characteristics of ZnO Nanoparticles Electron Transport Layer Improved by Intense-Pulsed Light Post-Treatment on Hole-Electron Injection Balance of Quantum-Dot Light-Emitting Diodes
Source: Materials (Basel). 2020 Nov 9;13(21):5041. doi: 10.3390/ma13215041 (PMC7664918; doi:10.3390/ma13215041)
Supplement: Supplementary file 1 [file materials-13-05041-s001.pdf]

materials

Supporting Information

Effect of Time-Dependent Characteristics of Zno Nanoparticles Electron Transport Layer Improved by Intense-Pulsed Light Post-Treatment on Hole-Electron Injection Balance of Quantum-Dot Light-Emitting Diodes

Young Joon Han [1,2], Kyung-Tae Kang [1], Byeong-Kwon Ju [2,*] and Kwan Hyun Cho [1,*]

[1] Manufacturing Process Platform Research and Development Department, Korea Institute of Industrial Technology (KITECH), 143 Hanggaul-ro, Sangnok-gu, Ansan-si 15588, Korea; youngjhan@kitech.re.kr (Y.J.H.); ktkang@kitech.re.kr (K.-T.K.)

[2] Department of Electrical and Electronics Engineering, College of Engineering, Korea University, 145 Anam-ro, Seongbuk-gu, Seoul 02841, Korea

* Correspondence: bkju@korea.ac.kr (B.-K.J.), khcho@kitech.re.kr (K.H.C.)

Received: 10 October 2020; Accepted: 5 November 2020; Published: date

1. Supplementary Materials

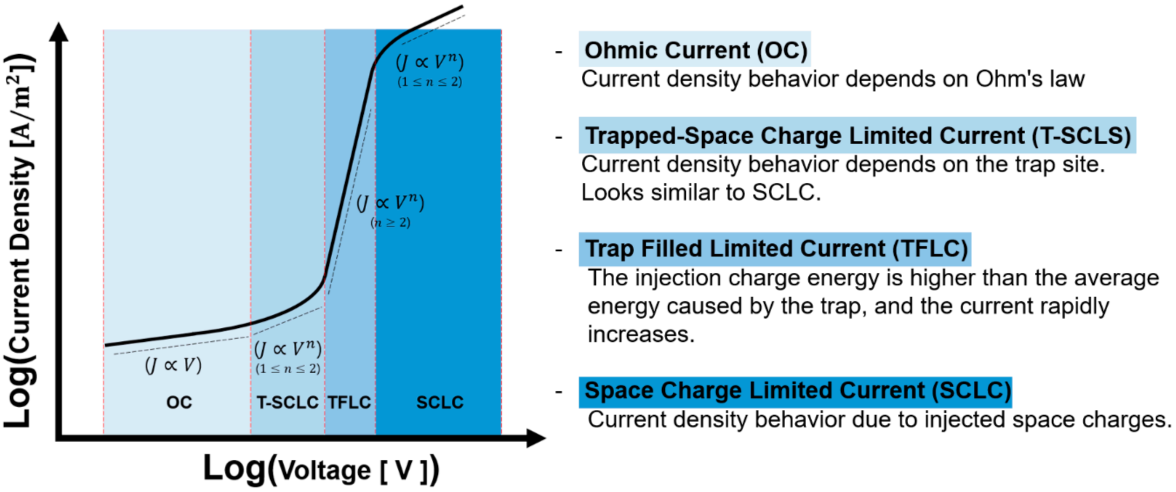

Figure S1. Typical log-log plot of the current density-voltage characteristics of QLEDs. Based on this theory of current behavior in the device, we conducted an 8-day measurement of the current density behavior of HODs and EODs, in addition to QLEDs; the effects of atmospheric oxygen and moisture on the device and the tendency of the ZnO NPs ETL characteristics during IPL post-treatment were analyzed.

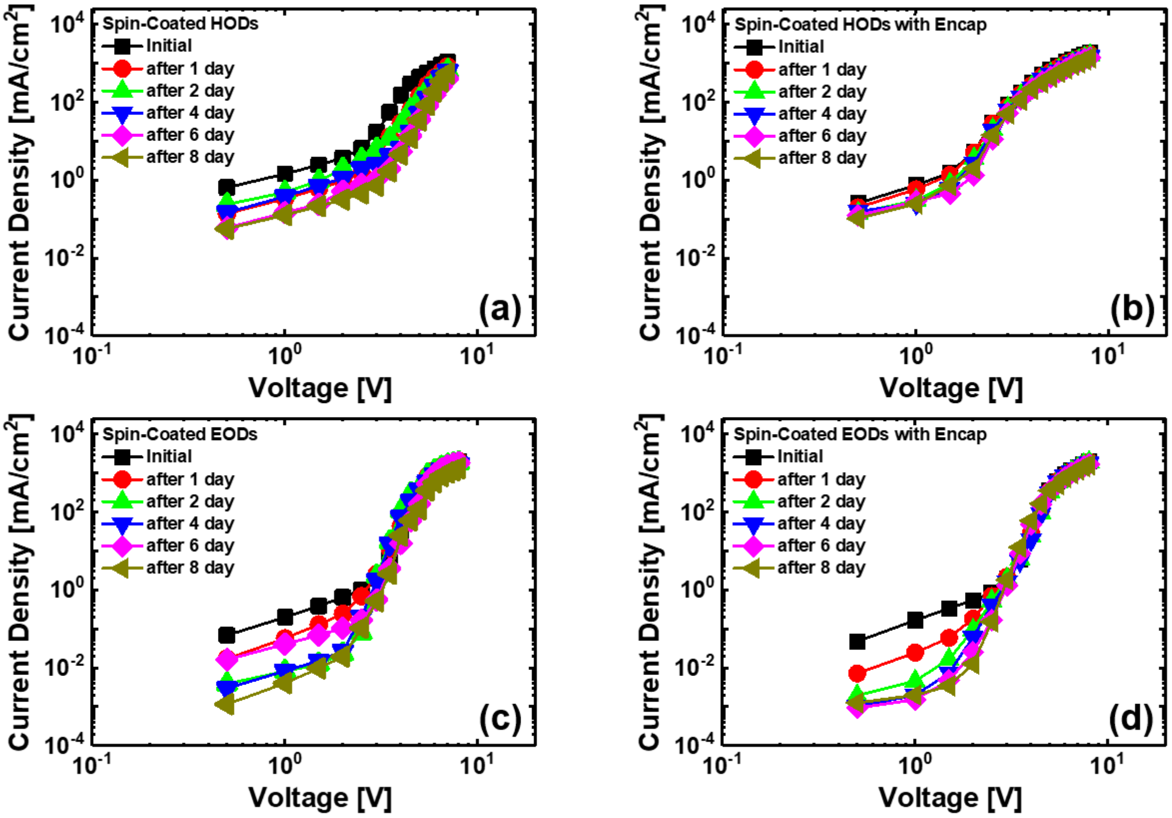

Figure S2. Current density-voltage characteristics of the HODs (**a**) with and (**b**) without encapsulation and the EODs (**c**) with and (**d**) without encapsulation. By encapsulation, in the HODs, deterioration of device performance due to moisture and oxygen due to organic materials was improved. In the EODs, decrease of current density in OC and T-SCLC regions over time was consistently improved.

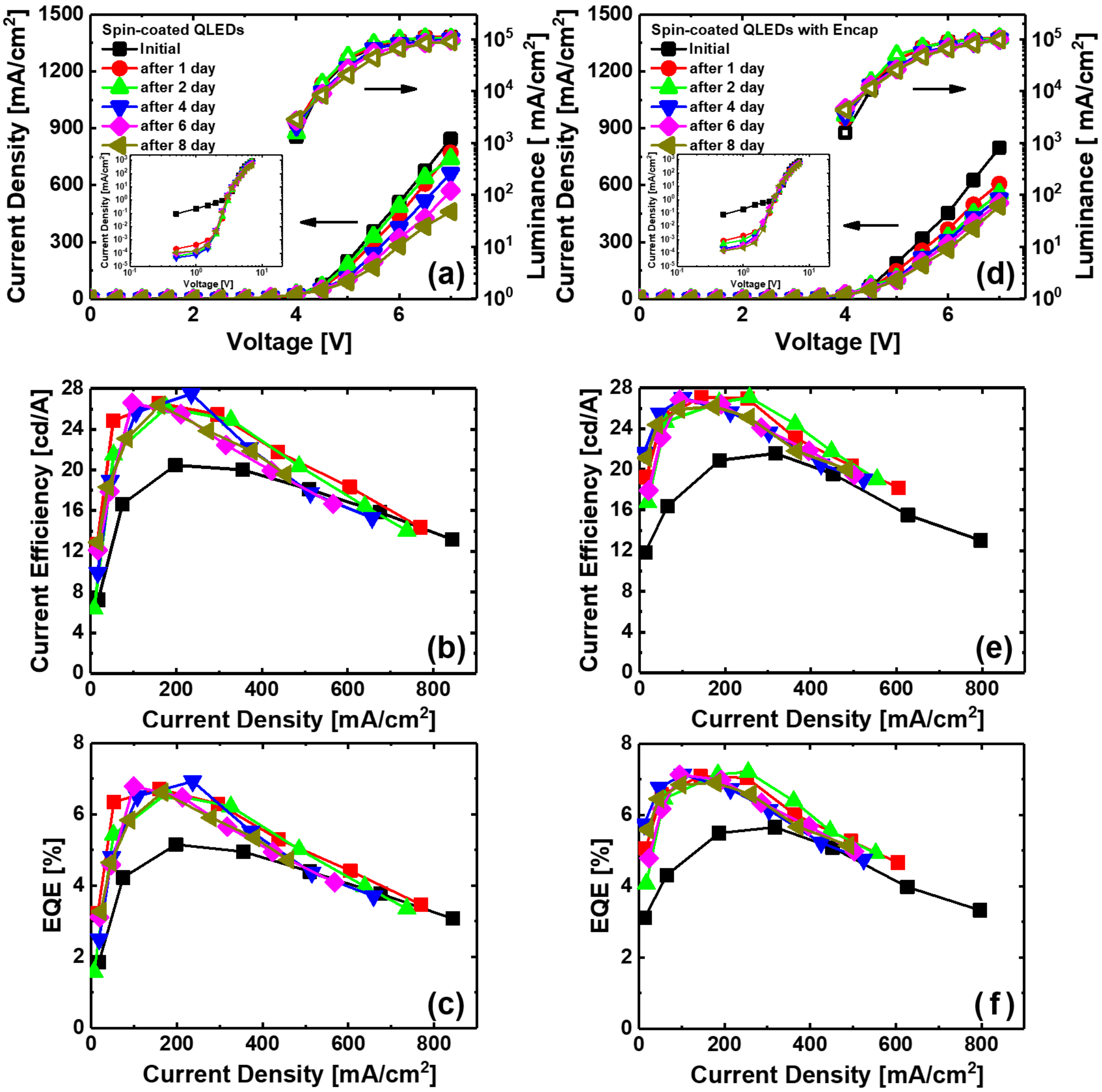

Figure S3. IVL characteristics of the spin-coated QLEDs (**a**–**c**) with and (**d**–**f**) without encapsulation (Inset of (**a**) and (**d**): log-log plot of the current density-voltage curve). QLEDs has a tendency to maintain a constant current density by encapsulation, and the efficiency curves is almost constant from the after 1 day.
